# Supplementary material for: Visualizing ultrafast photothermal dynamics with decoupled optical force nanoscopy
Source: Nat Commun. 2023 Nov 10;14:7267. doi: 10.1038/s41467-023-42666-9 (PMC10638245; doi:10.1038/s41467-023-42666-9)
Supplement: Supplementary file 1 — Supplementary Information [file 41467_2023_42666_MOESM1_ESM.pdf]

Supplementary information for **Visualizing Ultrafast Photothermal Dynamics with Decoupled Optical Force Nanoscopy**

Hanwei Wang[1,2], Sean M. Meyer[3], Catherine J. Murphy[3], Yun-Sheng Chen[1,2,4,5,6], Yang Zhao[1,2,4,5,7]*

[1] Department of Electrical and Computer Engineering, University of Illinois Urbana-Champaign, Urbana, IL, USA.

[2] Nick Holonyak Micro and Nanotechnology Laboratory, University of Illinois Urbana-Champaign, Urbana, IL, USA.

[3] Department of Chemistry, University of Illinois Urbana-Champaign, Urbana, IL, USA.

[4] Beckman Institute for Advanced Science and Technology, University of Illinois Urbana-Champaign, Urbana, IL, USA.

[5] Department of Bioengineering, University of Illinois Urbana-Champaign, Urbana, IL, USA.

[6] Department of Biomedical and Translational Sciences, Carle Illinois College of Medicine, University of Illinois Urbana-Champaign, Urbana, IL, USA

[7] Carl R. Woese Institute of Genomic Biology, University of Illinois Urbana-Champaign, Urbana, IL, USA

Supplementary Note 1: Experimental setup

The setup of the Dofn system is shown in Supplementary Fig. 1. A supercontinuum laser (NKT Photonics SuperK EXTREME) filtered by a tunable filter (NKT Photonics SuperK VARIA) is used as the source with a center wavelength of 600 nm and 700 nm and a bandwidth of 10 nm. The laser is modulated with an acousto-optic modulator (AOM) (Gooch & Housego AOMO

3080-125) controlled by a function generator (Agilent 33250A 80 MHz Function/Arbitrary Waveform Generator) with a modulation frequency between 160 kHz to 180 kHz according to the cantilever's resonance frequency. The AOM has a rise and fall time of between 23 ns and 65 ns, which is small compared to the thermal relaxation time of 283 ns. The slightly non-sharp rising and falling edges will result in a minor phase increase for both the optical gradient force and the photothermal force. However, as both of the optical forces will experience such a phase change, the relative phase relationship between them will be mostly unchanged. The output laser is unpolarized initially and coupled to the AFM system through a multi-mode optical fiber. At the output of the fiber, the laser is collimated and polarized to circularly polarized light (CPL) with a polarizing beam splitter and a quarter wave plate. The laser power focused on the sample measured to be 8.59 mW at 700 nm and 2.38 mW at 600 nm. The AFM (Oxford Asylum MFP-3D-BIO) is integrated with an inverted optical microscope (Olympus IX81) with a high NA oil-immersion objective lens (Olympus PlanApo 60x). The laser is focused on a spot with a diameter of around 50 μm. The AFM head is integrated with a built-in specialized bandpass filter (Semrock FF01-835/70) that blocks the laser from going to the photodetector. We use the AFM probe of 4XC-NN with the standard AC mode cantilever with a resonance frequency of around 170 kHz. To extract the optical forces, the deflection signal of the cantilever is demodulated with a lock-in amplifier (Signal Recovery 7280 DSP Lock-in Amplifier) with an integration time of 100 ms; the low-pass filter bandwidth is set to 10 Hz with a slope of 12 dB/Octave.

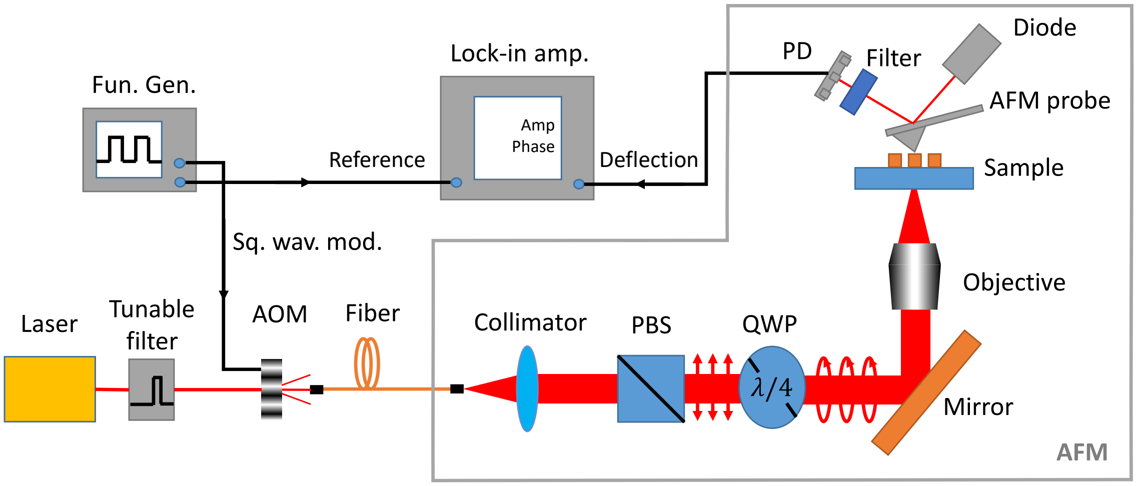

Supplementary Fig. 1| The schematic illustration of the Dofn system setup. The AFM region indicates the temperature and vibration control chamber of AFM. Fun. Gen.: function generator; Sq. wav. mod: square wave modulation; AOM: acoustic optical modulator; amp: amplifier; Amp: amplitude; PBS: polarizing beam splitter; QWP: quarter wave plate; PD: photodetector.

All the measurements are done in a 10 µm-diameter window fabricated on a glass slide with a thickness of around 150 µm (Supplementary Figs. 2**b** and 2**c**). The window can be covered by the AFM tip during the optical force measurements. So, the laser leakage to the photodetector is significantly reduced. The window is fabricated with photolithography (using LOR 5B and AZ 5214 as a dual layer-photoresist), electron beam deposition of 200 nm of nickel, and lift-off. We spin coat and cure 200 nm of PMMA to the glass slide. Finally, gold nanorods are drop coasted to the glass slide (Supplementary Fig. 2**a**). The optical force is scanned inside the optical window and with the laser aligned to the AFM tip.

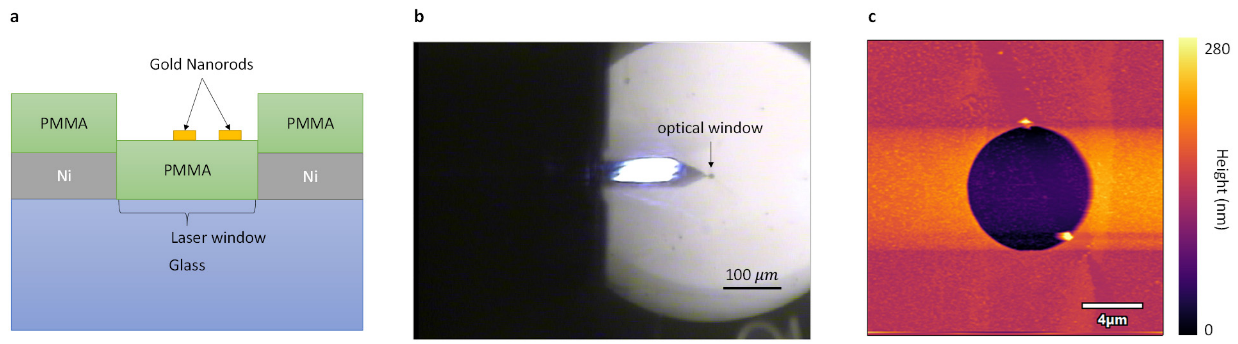

Supplementary Fig. 2| The optical window. a, The cross-section of the sample slides. A 10 μm-diameter optical window is fabricated on the glass slides with photolithography, E-beam deposition of 200 nm of nickel, and lift-off. 200 nm of PMMA is spin-coated and cured on top of the Ni-layer. Finally, gold nanorods are deposited on the sample slides through drop casting within the optical window and dried in a vacuum oven at room temperature. **b,** A top-view microscopy picture of the sample slide with an AFM tip engaged. During optical force measurements, the AFM tip is aligned with the bottom illumination laser. **c,** The AFM image of the optical window.

Supplementary Note 2. Optical gradient force simulation

In the main text equation (1), we model the AFM tip as a small particle for optical gradient force with a dipole approximation. One may argue that this is not generally true because the size of the AFM probe is significantly larger than the wavelength. However, such an approximation remains valid when only the tip of the AFM probe is involved in the near-field interaction. To validate this approximation, we proceed with a full wave numerical simulation using the COMSOL Electromagnetics module. In the simulation, we used an AFM probe with a height of 500 nm. The simulated polarization density of the AFM probe and the nanorod is shown in Supplementary Fig. 3. The proportion of the AFM probe's non-zero polarization density is less

than 25 nm, as shown in Supplementary Fig. 3. Consequently, if the AFM probe is modeled as a dipole, the error should not be substantial.

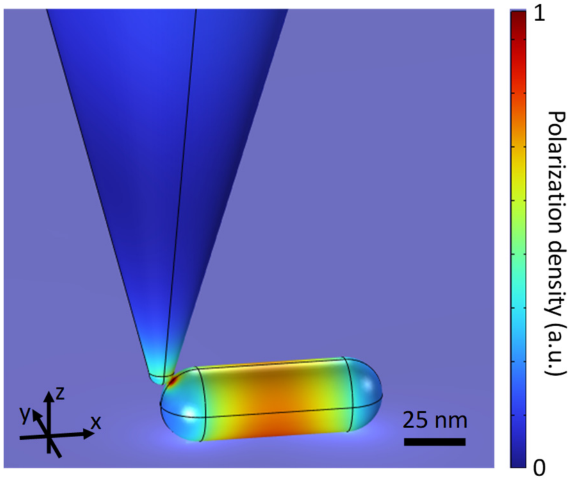

Supplementary Fig. 3| Simulated polarization density of the AFM probe and the nanorod. The AFM probe is made of silicon and has a tip radius of 5 nm, a height of 500 nm, and an aspect ratio (height/diameter) of 1.75. The tip of the AFM probe is kept 1 nm away from the surface of the nanorod.

We further simulate the 1D distribution of the optical force with the scanning line marked in Supplementary Fig. 4**a**. We simulate the optical gradient force by the surface integral of the Maxwell stress tensor on the AFM probe ass

$$\mathbf{F} = \oint_{probe} \boldsymbol{\sigma} d\mathbf{s} \,, \quad (1)$$

where σ is the Maxwell stress tensor on the AFM probe,

$\boldsymbol{\sigma} = \left[\varepsilon_0 \left(\mathbf{E} \otimes \mathbf{E} - \frac{1}{2}\mathbf{E}^2\mathbf{I} \right) + \frac{1}{\mu_0} \left(\mathbf{H} \otimes \mathbf{H} - \frac{1}{2}\mathbf{B}^2\mathbf{I} \right) \right]$, where $\mathbf{E}$ and $\mathbf{H}$ are the electrical and

magnetic fields, $\otimes$ represents a dyadic product, and $\mathbf{I}$ represents a 3-by-3 identity matrix. The simulated force distribution shown in Supplementary Fig. 4**b** fits well with the measured and theoretical distribution shown in Figures 2**f** and 2**i** in the main text.

The dominant effect in generating the optical gradient force differs for dielectric samples [1] and plasmonic samples. For the dielectric samples, the force is mainly given by the lightning rod effect of the AFM tip apex and its induced dipole moments on the substrate that are related to the dielectric properties of the substrate. On the other hand, for plasmonic samples, the optical force is mainly given by the localized electric field strength induced by the sample due to the plasmonic resonance.

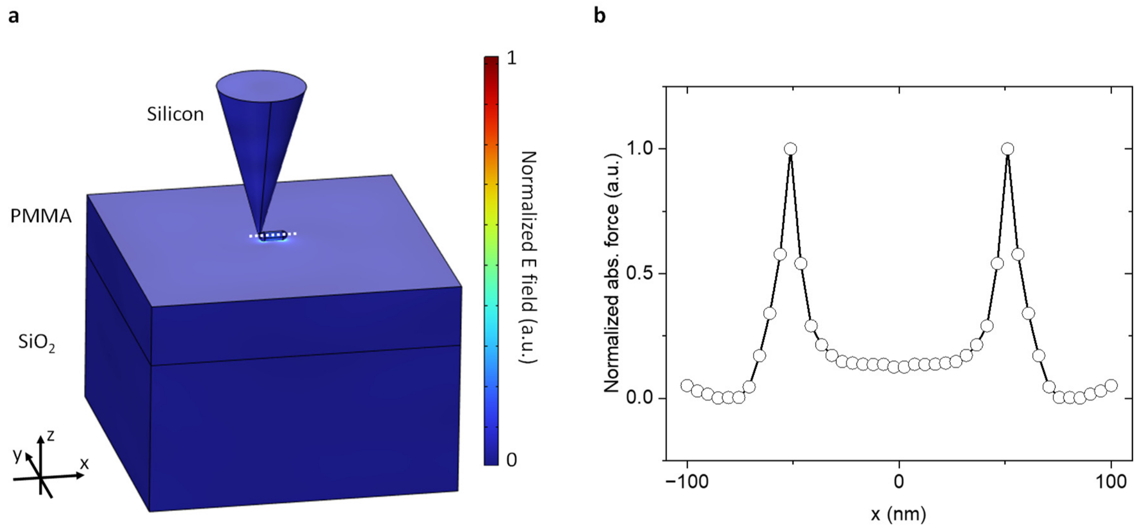

Supplementary Fig. 4| Simulation of the optical gradient force. **a,** Simulation model. The gold nanorod is placed on top of a substrate composed of a 200 nm PMMA layer and a 500 nm SiO_2 layer. The AFM tip is kept 1 nm away from the sample surface. The scanning line is marked as the dashed line. **b,** Normalized force distribution in the 1D scanning line marked in **a**.

Supplementary Note 3: Photothermal simulation

Here our sample is chemically synthesized gold nanorods. The thermal expansion of a single gold nanorod is simulated using finite element method (COMSOL Multiphysics, using the Electromagnetics and Heat Transfer modules) [2-5]. We first simulate the absorption cross section of the gold nanorod with COMSOL Electromagnetics module. Then, we simulate the temperature distribution with the AFM tip engaged to the sample surface with COMSOL Heat Transfer module, treating the nanorod as a heat source. The laser intensity is set as $1.18\times10^{16}\ W/m^2$. The averaged elevated temperature of the nanorod is calculated by $\langle \Delta T \rangle_{NP} = \int_{NP} \Delta T(\mathbf{r}) d\mathbf{r} / V_{nanorod}$, where NP represents the nanorod domain, $V_{nanorod}$ is the volume of the nanorod. f_{opt} is around 170 kHz, which gives a modulation period of 5.88 μs. As a result, the laser off-time is 4.41 μs. As shown in Supplementary Fig. 5**a**, the elevated temperature decreases to be <0.005k in 2.21 μs after the laser is off. As the elevated temperature of the nanorod drops to be significantly lower than its peak value of 10.9 K, simulation of a single period is sufficient. The averaged elevated temperature of the nanorod is shown in Supplementary Fig. 5**a**. As the thermal conductivity of the PMMA is significantly higher than gold, the nanorod takes 282.9 ns to reach 90% of its stationary temperature change, but the PMMA takes a longer time. That is the reason that the photothermal force map will still evolve after 300 ns in the laser on-period (Supplementary Figs. 5**c** and 5**d**). Please note that we define the thermal decay time as the time for the elevated temperature of the nanorod to reach 90% of its stationary temperature change (i.e., decay to 10% of its peak elevated temperature) with the square wave modulation in Supplementary Fig. 5**a**. Due to the absorption waveform of the nanorod is not of a short impulse, the thermal decay time here is different from the conventional definition [6].

The thermal field distribution at a different time is shown in Supplementary Figs. 5**b-d**. The photothermal force is proportional to the thermal expansion of the sample and AFM tip along the z-direction [1,2,7]. The photothermal is

$$F_{PT} \propto \sum_i \int_{PL} dl \beta_i(l) \Delta T(l) , \quad (2)$$

where PL represents the probing line, β_i is the thermal expansion coefficient of the substrate, the gold nanorod, and the AFM tip respectively. We assume the top of the AFM tip and the bottom of the PMMA substrate are held as constant temperatures and simulate the elevated temperature. The AFM tip is scanned across the sample area to acquire the map of the photothermal force.

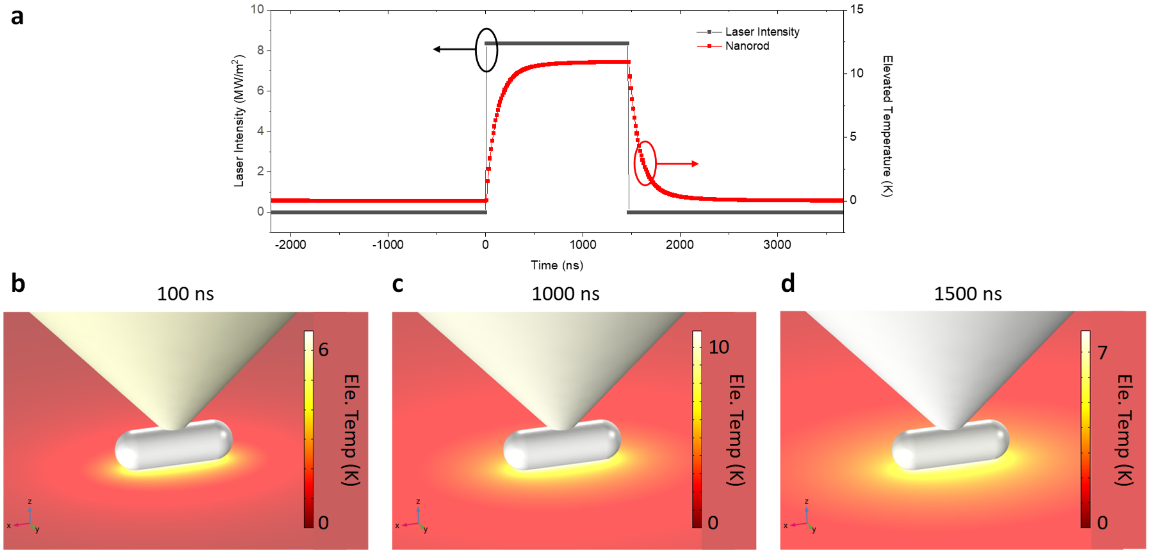

Supplementary Fig. 5| Simulation of the thermal dynamics. a, Simulated elevated temperature and laser's intensity as a function of time. The temperature of a gold nanorod is averaged over the simulation domain. Temperature distribution at **b,** 100 ns. **c,** 1000 ns, and **d,** 1500 ns.

The photothermal force is generated by the thermal expansion around the nanorod. In equation (2) of the main text, we simplify such an expansion as an isotropic displacement along the probing direction (dashed line along the z-direction as shown in Supplementary Fig 6**a**). Due to the irregular shape of the sample and the AFM tip, such a simplification may yield an error compared to the simulated photothermal expansion-induced displacement of the cantilever. As shown in Supplementary Fig. 6**a**, we simulate the thermal expansion induced by the heated nanorod with an elevated temperature of 10.9 K (close to the one shown in Figure 4**a** in the main text). Because the size of the nanorod (90 nm in length and 30 nm in width) is over three orders of magnitudes smaller than the AFM probe (170 μm in length and 40 μm in width), meshing the entire domain to achieve an accurate calculation will cause a significant computational burden. To reduce the computational burden and the meshing difficulty, we increase the size of the nanorod by 10 times but keep the temperature elevation the same as our previous calculation based on the actual nanorod dimension. We simulate the displacement of the cantilever using COMSOL Solid Mechanics Module (Supplementary Fig. 6**b**). The surrounding 4 surfaces of the substrate and the base of the cantilever are set as fixed boundaries. The temperature distribution along the probing direction is shown in Supplementary Fig. 6**c**.

We calculate the displacement with equation (2) and compare it with the simulated displacement (Supplementary Fig. 6**d**). The simulated displacement of the cantilever from the thermal expansion shows a difference from the calculated one given by equation (2) by less than 20%. The difference mainly comes from the non-isotropic expansion of the tip and the sample along the z-direction and would be smaller for the actual size because the heat will be spatially more confined. The spring constant of the cantilever was measured to be 8.6 N/m. By multiplying the spring constant and diving by the scale factor of 10, we get an approximate photothermal force of 70.8 pN, which roughly fits our measurement. The slightly bigger value

from the theoretical calculation may be attributed to the less confined temperature distribution because we used a larger rod in the simulation compared to the experiment.

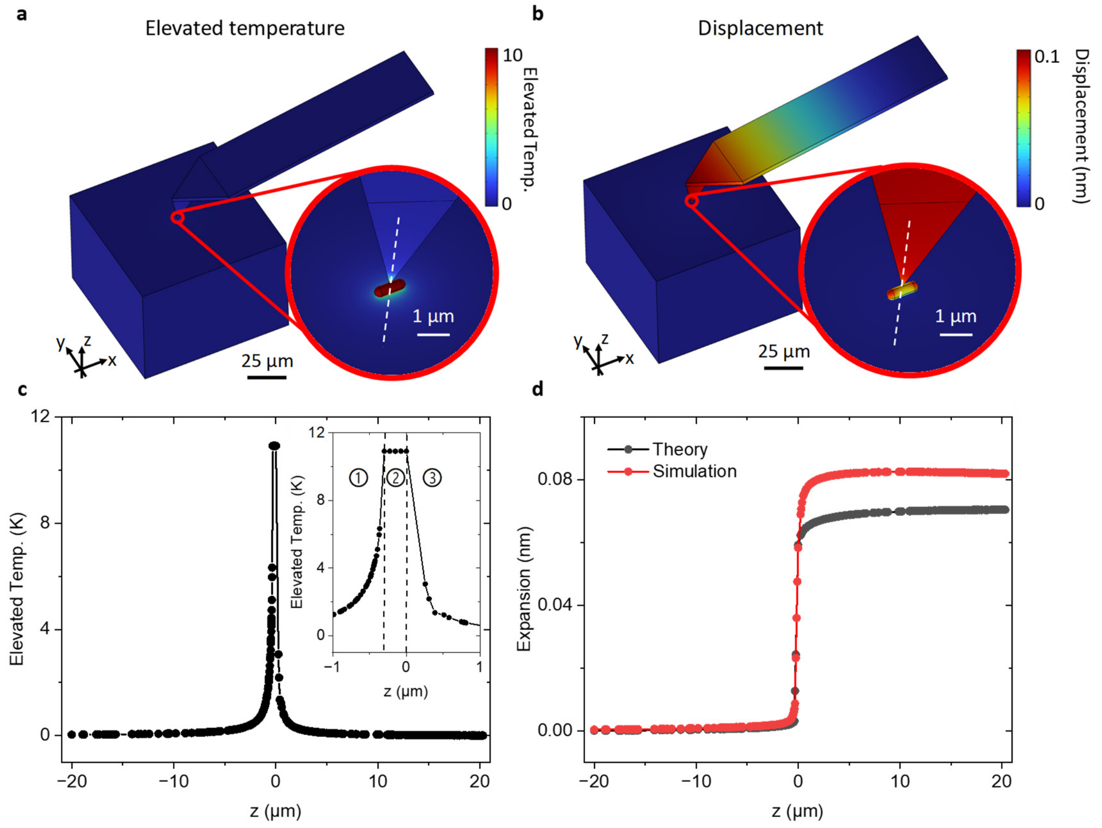

Supplementary Fig 6| Multiphysics simulation of the photothermal expansion. a, Simulated elevated temperature distribution. The elevated temperature of the nanorod is set as 10.9 K based on the simulation in Supplementary Fig. 5. We increase the nanorod by 10 times to a length of 0.9 μm, and a width of 0.3 μm, and simulate the substrate with a 50-μm-thick glass layer. **b,** Simulated displacement distribution. The probing direction is marked as the dashed line. **c,** Temperature distribution along the probing direction. The subplot shows the position of the substrate ①, nanorod ②, and AFM tip ③. d, Theoretical and simulated thermal expansion along the probing line. The theoretical curve is calculated by equation (2).

Supplementary Note 4: Photoacoustic simulation

The nanorod absorbs energy from light and generates acoustic pressure. The photoacoustic pressure, p, can be described with the following equation [8]:

$$(\nabla^2 - \frac{1}{v_s^2}\frac{\partial}{\partial t^2})p(\mathbf{r},t) = -\frac{\beta}{\kappa v_s^2}\frac{\partial^2 T(\mathbf{r},t)}{\partial t^2}, \tag{3}$$

where v_s is the acoustic speed, β is the thermal expansion coefficient, κ is the isothermal compressibility, $\kappa = \frac{C_p}{\rho v_s^2 C_v}$, C_p is the heat capacity at constant pressure, and C_v is the heat capacity at constant volume.

Using the Green function in free space $G(\mathbf{r},t,\mathbf{r'},t') = \frac{\delta(t-t'-\frac{|\mathbf{r}-\mathbf{r'}|}{v_s})}{4\pi|\mathbf{r}-\mathbf{r'}|}$. The photoacoustic pressure can be simplified as

$$p(\mathbf{r},t) = \frac{\beta}{4\pi\kappa v_s^2}\int d\mathbf{r'}\frac{1}{|\mathbf{r}-\mathbf{r'}|}\frac{\partial^2 T(\mathbf{r'},t')}{\partial t'^2}\Big|_{t'=t-\frac{|\mathbf{r}-\mathbf{r'}|}{v_s}} \ . \tag{4}$$

We first simulate the temperature with the transient thermal simulation discussed in the last section and calculate the photoacoustic pressure with the simulated temperature distribution $T(\mathbf{r'},t')$ according to equation (4). The normalized time-domain pressure in a period is shown in Supplementary Fig. 7**a**. The photoacoustic pressure acts on the AFM probe (Supplementary Fig. 7**b**) and results in the photoacoustic force [9,10]. The photoacoustic force calculated as the integration of the photoacoustic pressure over the probe's surface,

$$f_{PA}(t) = \int_{probe} d\mathbf{s}\cdot p(\mathbf{r},t) \ . \tag{5}$$

The intensity of the photoacoustic force's detected by the lock-in amplifier is calculated as

$$F_{PA} = \left\langle e^{i\omega_{opt}t} \mid f_{PA}(t) \right\rangle. \tag{6}$$

As the photoacoustic force is probed by a large area of the AFM probe, it is a non-localized force that is close to uniform in the nanoscale. As shown in Supplementary Fig. 7**c**, only some small variance can be observed with the field-of-view of 20 μm and the difference is ignorable (only 0.38%) with a field-of-view of around 150 nm.

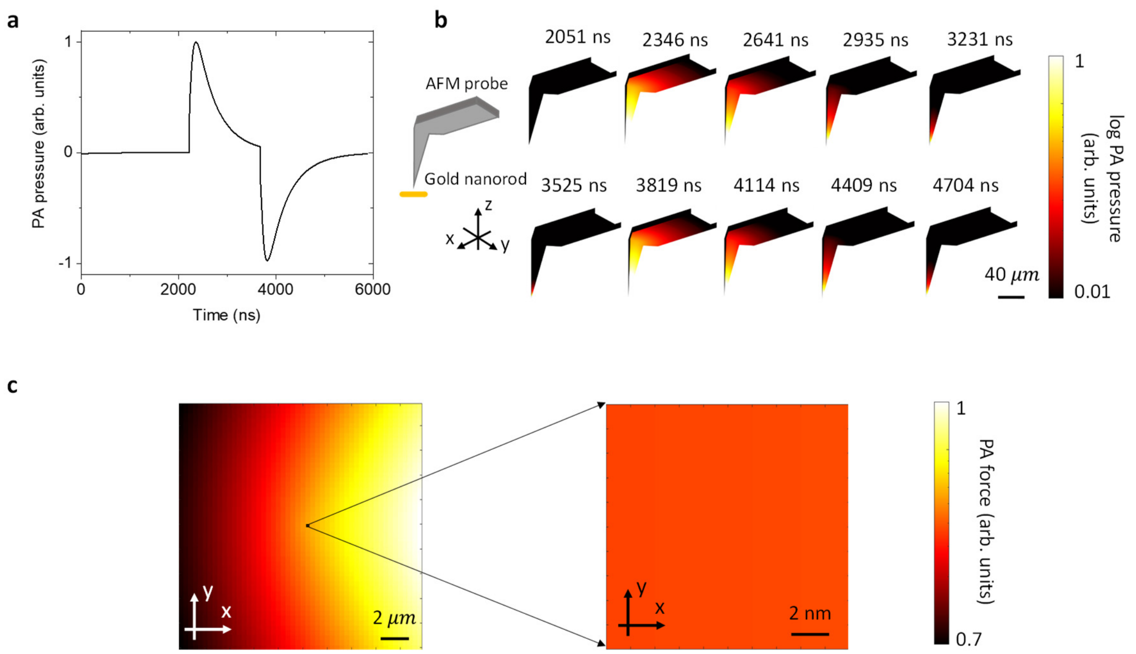

Supplementary Fig. 7| Photoacoustic simulation. a, Simulated photoacoustic pressure on the AFM probe in the time domain generated by the nanorod and its nearby surrounding media (PMMA, glass, and AFM tip). **b**, The time evolution of the photoacoustic pressure exerted on an AFM probe with the exact geometry matching the probe in use. **c**, Simulated spatial distribution of the photoacoustic force amplitude exerted on the AFM probe, and a zoomed-in distribution showing the force is relatively uniform across an area comparable to the AFM imaging field of view, validating the assumption that the photoacoustic force is a background force.

Supplementary Fig. 7**c** shows the photoacoustic force is a uniform background force. In the measurement, the total photoacoustic force is contributed by an ensemble of nanorods in the illumination area. To further understand the order of magnitude of the photoacoustic force and its temporal profile, we simulate the photoacoustic pressure given by equation (7) through the COMSOL PDE module. We use a uniform heat source that approximately matches the absorbed power of nanorods in the illumination area, where the incident light is modulated with a square wave with a peak intensity of 5 MW/m^2, as shown in Supplementary Fig. 8**a** (dashed curve, right y-axis). The AFM probe is modeled the same as the one used in the experiment (OPUS 4XC-NN, standard AC mode cantilever). We calculate the photoacoustic force by integrating the photoacoustic pressure over the surface area of the AFM probe:

$$\mathbf{F_{PA}} = \oiint_{probe} P d\mathbf{s}\,, \tag{7}$$

where P is the photoacoustic pressure on the AFM probe surface. As shown in Supplementary Fig. 8**a**, the simulated photoacoustic force shows two opposite peaks following the rising and falling edges of the square wave modulation as expected.

The pressure distribution at the peak position shows that the force is mainly probed by the sample-side surface of the cantilever (Supplementary Fig. 8**b**). The distortion of the waveform is attributed to the acoustic wave scattering by the AFM probe. The simulated photoacoustic force shows odd symmetry with respect to the center of the square wave, as illustrated by the phase relationship of the three types of optical forces in Figure 1 in the main text. The simulated PA force shows a peak intensity of 1.39 nN and a full width at half maximum of 79 ns. The average force in a period of around 5.9 μs is 37.2 pN. This estimation is close to our measured background force in Figure 2**b** of around 50 pF. The relatively lower value of the simulation may be attributed to that we ignored the photoacoustic force from the absorption of the AFM probe

and the PMMA film, which has a relatively high thermal expansion coefficient.

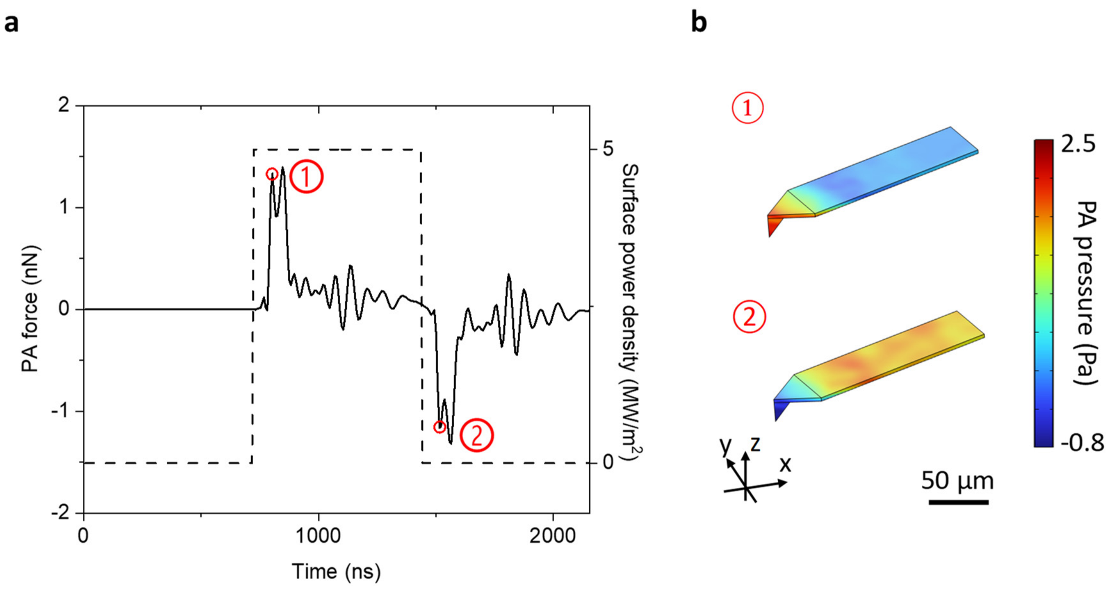

Supplementary Fig. 8| Multiphysics simulation of the photoacoustic force. a, Simulated photoacoustic force. The solid curve in the left plot represents the simulated photoacoustic force, F_{PA}, and the dashed curve represents the surface power intensity. **b,** Simulated photoacoustic pressure distribution. ① and ② correspond to the peak positions marked in **a**.

Supplementary Note 5: Optical gradient force and its dependency on the polarization

The optical gradient force is given by

$$\mathbf{F}_{\mathbf{G}} = \int_{tip} \mathbf{p}_{tip} \cdot \nabla \mathbf{E} dv , \tag{8}$$

where ∇ denotes the gradient of the corresponding field vectors. The dipole moment of the AFM tip is induced by the external electrical field, $\mathbf{p}_{tip} = \alpha_{tip}\mathbf{E}$, where α_{tip} is the polarizability of the tip. The AFM probe can mainly measure the force in the z direction. The measured optical

gradient force is therefore $F_G = \iiint_{tip} \alpha_{tip} E_z \frac{\partial E_z}{\partial z} dxdydz$. Thus, we get

$$F_G = \frac{1}{2}\sigma_{tip}\alpha_{tip}E_z^{\ 2} \tag{9}$$

where σ_{tip} is the cross section of the tip along the xy plane.

The distribution of the optical gradient force shows the electrical field distribution around the nanorod. As shown in Supplementary Fig. 9, the electrical field distribution is dependent on the circular polarization and wavelength of the incident light [2,11-13]. When the wavelength is 700 nm, mainly the transversal mode (plasmonic oscillation along the nanorod) is excited, the electrical field is strongest on both ends of the nanorod and the angular orientation of the electrical field around the nanorod is insignificant (Supplementary Figs. 9**b** and 9**d**). On the other hand, when the wavelength is 600 nm, both the transversal mode and the longitudinal mode are excited. The angular orientation of the electrical field is dependent on the handedness of the circularly polarized light. For left circularly polarized light in Supplementary Fig. 9**a**, electrical is rotated toward counterclockwise direction; and for right circularly polarized light in Supplementary Fig. 9**c**, the electrical field is rotated toward clockwise direction. The measured optical gradient force maps in Supplementary Figs. 9**e**-**h** fit well with the theoretical predictions.

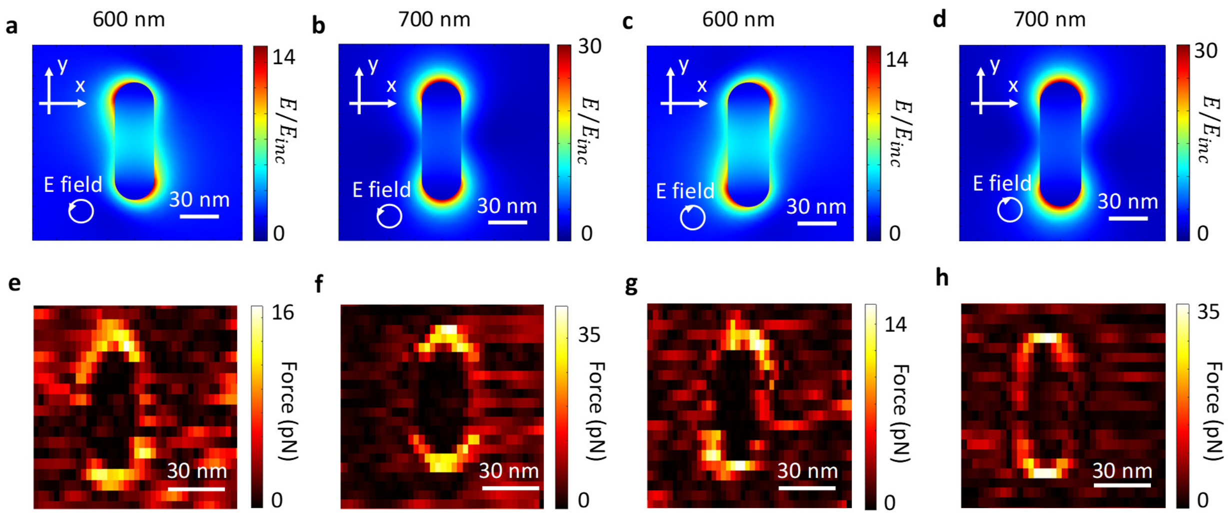

Supplementary Fig. 9| Decoupled optical gradient force under circularly polarized light (CPL) illumination. The electrical field distribution with **a,** left-handed CPL illumination at the wavelength of 600 nm; **b,** left-handed CPL at 700 nm; **c,** right-handed CPL at 600 nm; and **d,** right-handed CPL at 700 nm. **e- h,** Measured decoupled optical gradient force map with the same condition as Supplementary Figs. 9**a-d**.

Supplementary Note 6: Observation of the back-action of the photothermal expansion

Back-action of the photothermal expansion is the reason of the time-frame sensitivity photothermal force measurement [14,15]. To validate our theory, we proceed with the following experiment. As shown in Supplementary Fig. 10**a**, we fixed the laser's AC intensity and change its DC intensity. The tip will reach different equilibrium point in the force curve as shown in Supplementary Fig. 10**a**, corresponding to different k_{PT} . A higher laser's intensity results in a lower magnitude of the spring constant (being negative), and thus, a higher resonance frequency of the cantilever and a higher quality factor (Supplementary Fig. 10**b**). In the experiment, we fixed the piezo's modulation frequency as $f_d - f_0 = 0.5kHz$ and scan laser's

modulation frequency f_{opt}. We record the lock-in's signal and normalize its amplitude with the AC intensity of the laser measured with a photodiode (Newport Model 818-BB-21). As shown in Supplementary Figs. 10**c** and 10**d**, a higher laser's intensity results in a higher resonance frequency and broader bandwidth of the peak. The DC intensity is measured with a power meter (Newport Model 2936-R). The trend fits with the qualitative prediction of our theory.

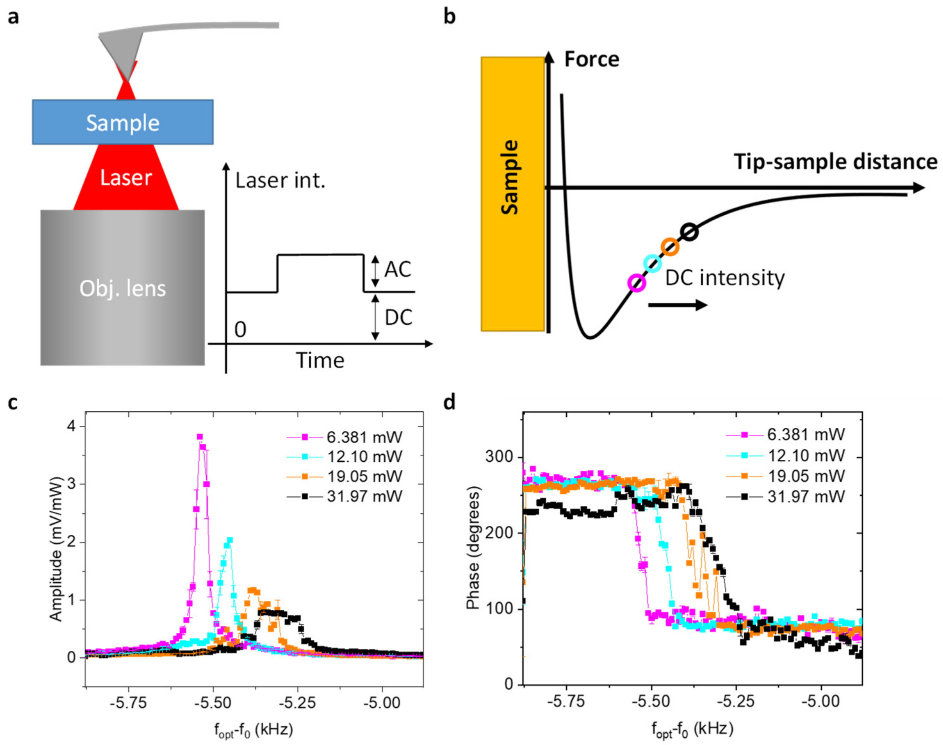

Supplementary Fig. 10| Frequency response of the optical force at different laser DC intensity to mimic the thermal expansion. a, Schematics of the laser modulation. **b**, Schematics of the force curve and the equilibrium points with different laser intensities. **c**, Amplitude, and **d**, phase of the lock-in amplifier's output normalized by the laser's AC intensity driven by the photothermal force, showing a frequency shift of the mechanical resonance. The error bars indicate the standard deviation, and the data points indicate the mean values of three measurements.

Supplementary Note 7: Theory of phase tunability of the Dofn system

The Dofn system can be modeled with the following time-domain equation [9,16]:

$$m\ddot{z} + m\gamma\dot{z} + kz = F_d + F_{opt} + F_{int} , \tag{10}$$

where m is the effective mass of the cantilever, γ is the damping coefficient, which comes from the viscosity of the sample and the air resistance to the cantilever and assumed to be a constant. k is the spring constant of the cantilever. F_d is the driving force of the piezo, which has a frequency dependence of ω_d , $\omega_d = 2\pi f_d$; F_{opt} is the overall optical-induced force; F_{int} describes the tip-sample interaction. Here, we assume a linear tip-sample force, $F_{int} = -k_{sp}z$, where k_{sp} describes an effective spring constant in addition to the spring constant of the cantilever.

The free-air deflection of the cantilever, which is measured to be around 110 nm in the experiment, is mainly controlled by the driving force F_d and its frequency f_d.

$$H^{(0)}\left|Z_{Free}\right\rangle = F_d e^{i\omega_d t} , \tag{11}$$

where $H^{(0)} = m\frac{d^2}{dt^2} + m\gamma\frac{d}{dt} + k$ is the transfer function with the laser off and when the AFM tip is not engaged, $\omega_d = 2\pi f_d$. Therefore, $\left|Z_{Free}\right\rangle = \frac{F_d}{-m\omega_d^{\;2} + im\omega_d\gamma + k} e^{i\omega_d t}$.

When the cantilever is engaged, the deflection of the cantilever is

$$H^{(1)}\left|Z_{Engaged}\right\rangle = F_d e^{i\omega_d t} , \tag{12}$$

where $H^{(1)} = m\frac{d^2}{dt^2} + m\gamma\frac{d}{dt} + k + k_{sp}$. Therefore, $\left|Z_{Engaged}\right\rangle = \frac{F_d}{-m\omega_d{}^2 + im\omega_d\gamma + k + k_{sp}} e^{i\omega_d t}$, which is reduced by the tip-sample interaction.

$$\left\langle Z_{Engaged} \mid Z_{Engaged} \right\rangle = \xi^2 \left\langle Z_{Free} \mid Z_{Free} \right\rangle, \tag{13}$$

where ξ is the engagement factor. By solving equation (11)-(13), we get two possible k_{sp},

$$k_{sp}^{\pm} = -k + m\omega_d{}^2 \pm \frac{1}{\xi}\sqrt{(k - m\omega_d^2)^2 + (1-\xi^2)(m\gamma\omega_d)^2}, \tag{14}$$

k_{sp}^{+} corresponds to repulsive mode and k_{sp}^{-} corresponds to attractive mode.

When the laser is on, the optical-induced forces are considered as a perturbation to the system. The deflection at the laser's modulation frequency f_{opt} follows

$$H^{(1)}\left|Z_{opt}\right\rangle = F_{opt}e^{i\omega_{opt}t}, \tag{15}$$

where $\omega_{opt} = 2\pi f_{opt}$.

Therefore,

$$\left\langle e^{i\omega_{opt}t} \mid Z_{opt}^{\pm} \right\rangle = \frac{F_{opt}}{-m\omega_{opt}^2 + im\gamma\omega_{opt} + m\omega_d^2 \pm \frac{1}{\xi}\sqrt{(k - m\omega_d^2)^2 + (1-\xi^2)(m\gamma\omega_d)^2}}, \tag{16}$$

$\left|Z_{opt}^{+}\right\rangle$ is given by the repulsive mode and $\left|Z_{opt}^{-}\right\rangle$ is given by the attractive mode. In the experiment, we use a 70% amplitude as the set point for the engagement, which means the cantilever's engaged amplitude is locked at 70% of its free air amplitude, $\xi = 0.7$. The cantilever

has a mechanical resonance frequency of 174.1 kHz, a spring constant of 8.6 N/m, and a damping coefficient of $5.65\times10^{3}s^{-1}$, which are all measured quantities. The amplitude and phase of the optical induced forces are a function of both the piezo dithering frequency f_d and the laser modulation frequency f_{opt}.

In repulsive or attractive modes, we plot the theoretical frequency-dependent map of the amplitude and phase of the optical induced forces, as shown in Supplementary Fig. 11. The energy of the cantilever of the two modes are $E_{Z_{opt}^{\pm}}=\frac{1}{2}(m\omega_d^2\pm\frac{1}{\xi}\sqrt{(k-m\omega_d^2)^2+(1-\xi^2)(m\gamma_{sys}\omega_d)^2})\langle Z_{opt}^{\pm}|Z_{opt}^{\pm}\rangle$. As the attractive mode is at a lower energy state, a stronger signal intensity can be obtained with the same amount of power.

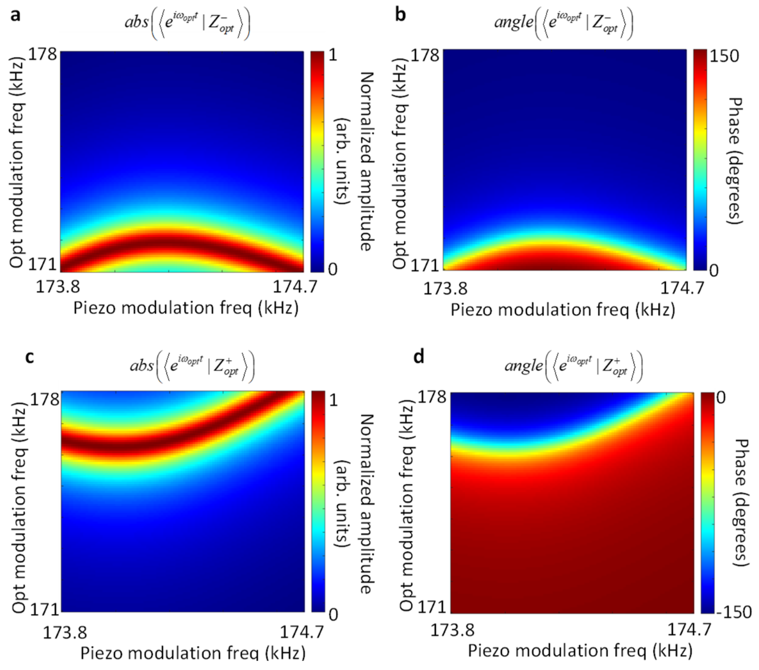

Supplementary Fig. 11| Theoretical frequency responses of the cantilever's deflection signal at the optical modulation frequency. a, Amplitude and **b,** phase of the Z_{opt}^{-} mode. **c,** Amplitude and d, phase of the Z_{opt}^{+} mode.

Supplementary Note 8: Temporal resolution of the Dofn system

The minimum detectable temporal resolution is determined by the jittering of the cantilever's deflection given by the fluctuation of the engagement factor $\delta\xi$. The standard deviation of phase is measured to be 2.12 degrees (an overall 60 measurements with 20 different piezo modulation frequency as shown in Supplementary Fig. 12). The detection limit is estimated to be 32.7 nanoseconds.

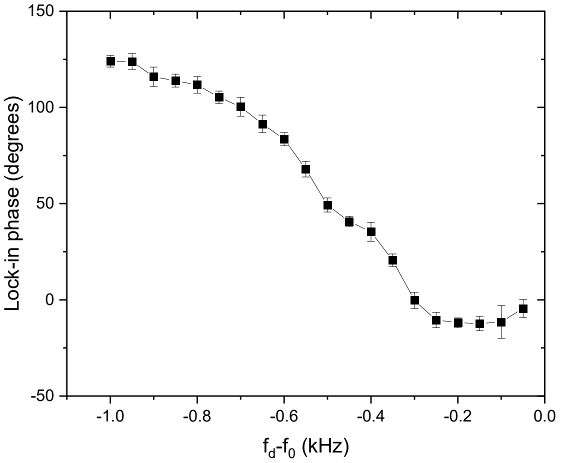

Supplementary Fig. 12| Measured phase at different piezo modulation frequencies. The optical frequency $f_{opt} - f_0$ is fixed at -1.5 kHz, the free air amplitude is around 110 nm, and the engagement factor is 70%. The error bars indicate the standard deviation, and the data points indicate the mean values of three measurements.

Supplementary Note 9: Sensitivity of the optical force measurement

The sensitivity of the measured optical induced forces is limited by the thermal noise of the cantilever's deflection and the sideband leakage of the deflection driven by the piezo (Supplementary Fig. 13**a**). The measurement is conducted at room temperature, the Brownian motion of the air molecules and the molecules on the sample surface results in the thermal

noise with an intensity of around 0.3 pN (Supplementary Fig. 13**b**). The measured spectrum of the thermal noise is shown in Supplementary Fig. 13**b**, which is a white noise being uniform at all frequency. As shown in Supplementary Figs. 13**c**-**e**, the noise due to the sideband leakage is more significant with a lower frequency difference between the laser's modulation frequency and the piezo's driven frequency $f_{opt} - f_d$. As the dose curve shown in Supplementary Fig. 9**f**, the detection limit varies from 0.5 pN to 10 pN depending on $f_{opt} - f_d$.

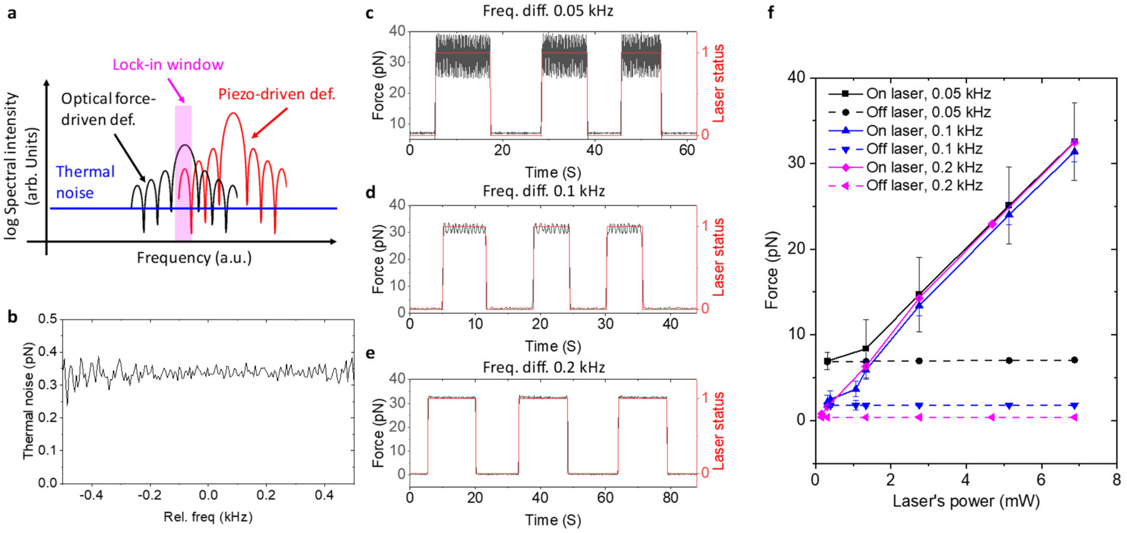

Supplementary Fig. 13| Sensitivity of the measured optical force. a, Schematics of the spectra of the optical force-driven deflection, the piezo-driven deflection, and the thermal noise. The sources of the optical force's noises compose of the thermal noise and sideband leakage of the piezo's driven peak. **b,** Measured thermal noise. The thermal noise is a white noise with the spectral power density of 0.31 pN at 15 °C. The x-axis is the relative frequency of the lock-in amplifier to the resonance frequency of the cantilever (168.944 kHz). On- and off-laser testing with a laser power of 6.9 mW, and the frequency difference between the laser's modulation frequency and the piezo's dithering frequency of **c**, 0.05 kHz, **d,** 0.1 kHz, and **e,** 0.2 kHz. **f,** The sensitivity measurement of optical forces as a function of laser power. The error bars indicate the standard deviation, and the data points indicate the mean values of three measurements.

Supplementary Note 10: Correction to the thermal drifting

We recorded the amplitude and phase of the optical force while scanning the sample (Supplementary Fig. 14**a**). Each recording of a force map typically takes around 4 minutes. As the maps shown in Fig. 14**b**, the nanorod will drift around 50 nm due to the thermal drift of the sample and/or the probe [17]. This drift can be corrected with post-processing assuming a constant drifting velocity. As shown in Supplementary Fig. 14**c**, after drift-correction, the nanorod shifts back to a vertical position. We then omitted the background data that exceeds the rectangular boundary in Supplementary Fig. 14, as well as Figures 2 and 4 in the main text.

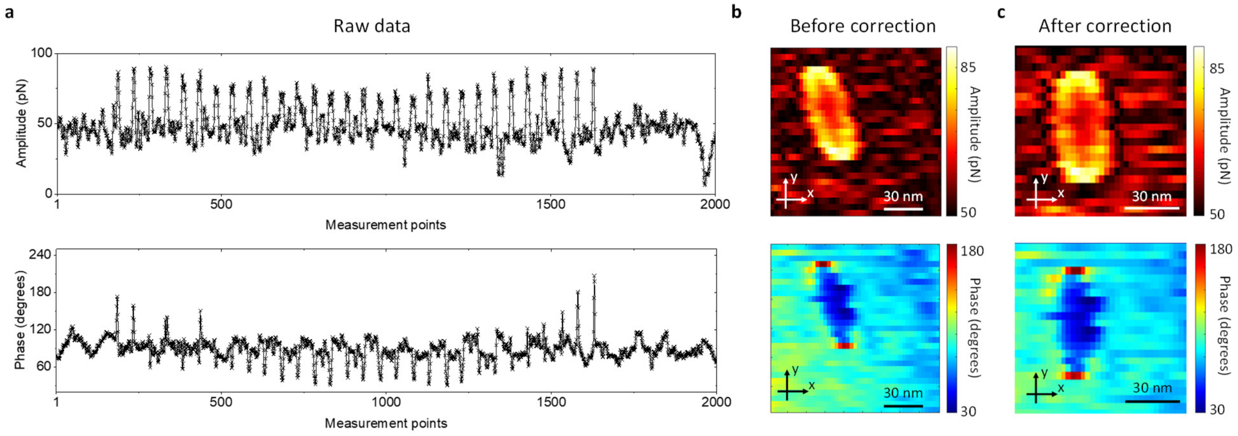

Supplementary Fig. 14| Correction of the thermal drift of the scanning probe. a, Raw data of amplitude and phase of the optical force. **b,** Maps of the amplitude and phase of the optical force before drift correction. **c,** Map of the amplitude and phase of the optical force after drift correction.

Supplementary Note 11: Thermal softening of the cantilever

To rule out the possibility of softening of the cantilever due to increasing DC laser power, we conducted additional experiments by measuring the resonance frequencies while changing the

DC laser power. Our experiments confirm that the shifted resonance is indeed from the back-action of the photothermal expansion instead of the heating and softening of the cantilever. As shown in Supplementary Fig. 15**a** below, we used a similar laser power as in Figure 3**a** in the main text and a clear glass slide as the sample. We measured the tuning curve of the cantilever as we increased the DC laser power and recorded the shift of the resonance frequency. Our new experiment shows that the shift of the resonance by such an effect is within 0.032 kHz.

We compare the experimental results when the tip is engaged to a nanorod (Supplementary Fig. 15**b**). Supplementary Fig. 15**c** shows that the shift of resonance due to the heating and softening of the cantilever is around one order of magnitude smaller and in opposite direction compared to the shift due to back-action. The significant difference in the frequency shift suggests that the changes in resonance in Dofn are mainly due to the back-action of the photothermal expansion, whereas the shift due to the DC laser power change is negligible.

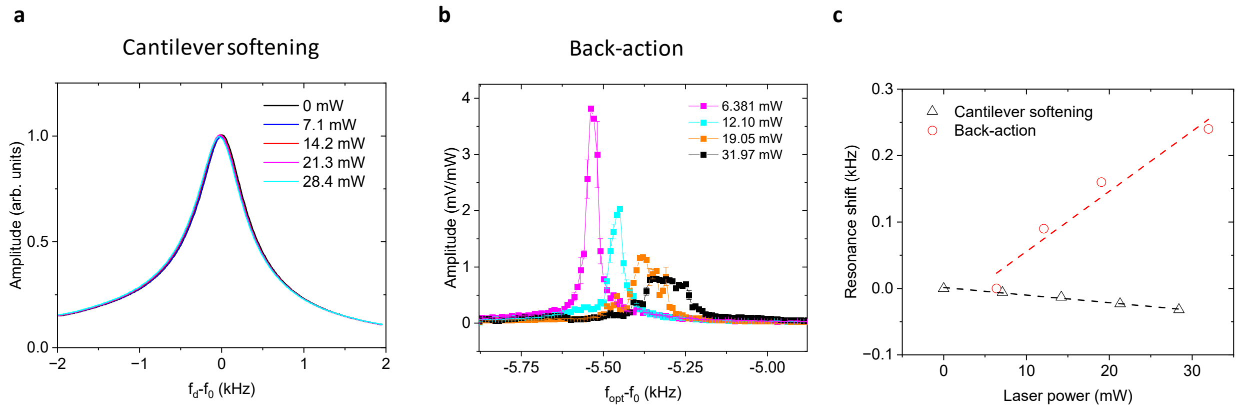

Supplementary Fig. 15| Shift of resonance frequency by heating and softening of the cantilever and back-action of the photothermal force. f_0 denotes the mechanical resonance frequency of the cantilever without laser. **a,** Tuning curve with different DC laser intensity. **b,** Oscillation at the laser modulation frequency with different AC laser intensities. The error bars indicate the standard deviation, and the data points indicate the mean values of three measurements. **c,** Frequency shift at different laser intensities of the two cases.

Supplementary References

1 Jahng, J., Potma, E. O. & Lee, E. S. Tip-enhanced thermal expansion force for nanoscale chemical imaging and spectroscopy in photoinduced force microscopy. *Analytical chemistry* **90**, 11054-11061 (2018).

2 Lahiri, B., Holland, G., Aksyuk, V. & Centrone, A. Nanoscale imaging of plasmonic hot spots and dark modes with the photothermal-induced resonance technique. *Nano letters* **13**, 3218-3224 (2013).

3 Ekici, O. *et al.* Thermal analysis of gold nanorods heated with femtosecond laser pulses. *Journal of physics D: Applied physics* **41**, 185501 (2008).

4 Chen, Y.-S., Zhao, Y., Yoon, S. J., Gambhir, S. S. & Emelianov, S. Miniature gold nanorods for photoacoustic molecular imaging in the second near-infrared optical window. *Nature nanotechnology* **14**, 465-472 (2019).

5 Baffou, G. & Quidant, R. Thermo‐plasmonics: using metallic nanostructures as nano‐sources of heat. *Laser & Photonics Reviews* **7**, 171-187 (2013).

6 Chen, X., Chen, Y., Yan, M. & Qiu, M. Nanosecond photothermal effects in plasmonic nanostructures. *ACS nano* **6**, 2550-2557 (2012).

7 Lu, F., Jin, M. & Belkin, M. A. Tip-enhanced infrared nanospectroscopy via molecular expansion force detection. *Nature photonics* **8**, 307-312 (2014).

8 Wang, L. V. & Wu, H.-i. *Biomedical optics: principles and imaging*. (John Wiley & Sons, 2012).

9 Almajhadi, M. A., Uddin, S. M. A. & Wickramasinghe, H. K. Observation of nanoscale opto-mechanical molecular damping as the origin of spectroscopic contrast in photo induced force microscopy. *Nature communications* **11**, 1-9 (2020).

10 O'Callahan, B. T., Yan, J., Menges, F., Muller, E. A. & Raschke, M. B. Photoinduced tip–sample forces for chemical nanoimaging and spectroscopy. *Nano Letters* **18**, 5499-5505

(2018).

11 Imura, K., Nagahara, T. & Okamoto, H. Near-field optical imaging of plasmon modes in gold nanorods. *The Journal of chemical physics* **122**, 154701 (2005).

12 Imura, K., Nagahara, T. & Okamoto, H. Imaging of surface plasmon and ultrafast dynamics in gold nanorods by near-field microscopy. *The Journal of Physical Chemistry B* **108**, 16344-16347 (2004).

13 Tumkur, T. U. *et al.* Photoinduced force mapping of plasmonic nanostructures. *Nano letters* **16**, 7942-7949 (2016).

14 Ma, J. *et al.* Optical back-action on the photothermal relaxation rate. *Optica* **8**, 177-183 (2021).

15 Ma, J. *et al.* Photothermally induced transparency. *Science advances* **6**, eaax8256 (2020).

16 Metzger, C. H. & Karrai, K. Cavity cooling of a microlever. *Nature* **432**, 1002-1005 (2004).

17 Abe, M. *et al.* Drift-compensated data acquisition performed at room temperature with frequency modulation atomic force microscopy. *Applied physics letters* **90**, 203103 (2007).
